# METTL3-mediated m6A modification of *STEAP2* mRNA inhibits papillary thyroid cancer progress by blocking the Hedgehog signaling pathway and epithelial-to-mesenchymal transition

**DOI:** 10.1038/s41419-022-04817-6

**Published:** 2022-04-18

**Authors:** Yue Zhu, Xinzhi Peng, Qianlei Zhou, Langping Tan, Cheng Zhang, Shaojian Lin, Miaoyun Long

**Affiliations:** 1 Guangdong Province Key Laboratory of Malignant Tumor Epigenetics and Gene Regulation, Research Center of Medicine, Sun Yat-Sen Memorial Hospital, Sun Yat-Sen University, Guangzhou, 510120 China; 2 Department of Thyroid Surgery, Sun Yat-Sen Memorial Hospital, Guangzhou, 510120 China

**Keywords:** Metastasis, Prognostic markers

## Abstract

Papillary thyroid cancer (PTC) is a common endocrine system malignancy all over the world. Aberrant expression of six transmembrane epithelial antigen of the prostate 2 (STEAP2) has been functionally associated with cancer progression in many cancers. Nevertheless, its biological function in PTC is still unclear. Here, we found that PTC tissues had preferentially downregulated STEAP2 as compared with noncancerous tissues. Low STEAP2 expression correlated with aggressive clinicopathological characteristics and dismal prognosis in patients with PTC. We performed gain- and loss-of-function experiments, including cell proliferation assay (Cell Counting Kit-8 assay), EdU (5-ethynyl-2′-deoxyuridine) and colony formation assays, transwell migration, and invasion assays, and constructed a nude mouse xenograft tumor model. The results demonstrated that *STEAP2* overexpression inhibited PTC cell proliferation, migration, and invasion in vitro and inhibited lung metastasis and tumorigenicity in vivo. Conversely, silencing *STEAP2* yielded the opposite results in vitro. Mechanistically, bioinformatics analysis combined with validation experiments identified *STEAP2* as the downstream target of methyltransferase-like 3 (METTL3)-mediated N6-methyladenosine (m6A) modification. METTL3 stabilized *STEAP2* mRNA and regulated STEAP2 expression positively in an m6A-dependent manner. We also showed that m6A-mediated *STEAP2* mRNA translation initiation relied on a pathway dependent on the m6A reader protein YTHDF1. Rescue experiments revealed that silencing *STEAP2* partially rescued the tumor-suppressive phenotype induced by METTL3 overexpression. Lastly, we verified that the METTL3–STEAP2 axis functions as an inhibitor in PTC by suppressing epithelial–mesenchymal transition and the Hedgehog signaling pathway. Taken together, these findings strongly suggest that METTL3-mediated *STEAP2* m6A modification plays a critical tumor-suppressive role in PTC progression. The METTL3–STEAP2 axis may be a potential therapeutic molecular target against PTC.

## Background

The most predominant malignancy of the endocrine system is thyroid cancer (TC), with steadily growing occurrence and morbidity around the world [1]. The most prevalent thyroid malignancy histotype is papillary thyroid carcinoma (PTC), and its response to therapy and the prognosis are good [2]. Nevertheless, around 1 in 10 patients with aggressive PTC develop distant metastases or recurrences within 10 years [3]. Hence, understanding the PTC progression molecular mechanism and identifying effective therapeutic targets for PTC remains important.

The human STEAP (six transmembrane epithelial antigen of prostate) family comprises four cell surface membranes: STEAP1–4. The STEAPs have very similar structure and domain organization and play a physiological role as oxidoreductases, where they participate in absorbing and reducing iron and copper [4, 5]. Emerging evidence has shown that the STEAPs are involved in inflammation, cell growth, and differentiation [4, 6]. Moreover, developing studies have found that STEAPs have a dual role in cancer progression. STEAP1 is overexpressed in human cancer tissues and cell lines, including those of bladder, prostate, ovarian, colon tumors, and Ewing’s sarcoma [7]. Prostate cancer has aberrantly upregulated STEAP1, STEAP2, and STEAP4, playing an important oncogene function in tumor malignancy [8–10]. On the contrary, STEAP3 inhibits prostate cancer cell proliferation by stimulating p53 expression [11]. Despite their importance in cancer development, current research on STEAPs is in its initial stages, and their expression status and function in PTC are still obscure.

N6-methyladenosine (m6A) RNA modification is tremendously prevalent, functionally modulating the eukaryotic transcriptome to affect mRNA export, splicing, translation, localization, and stability [12, 13]. m6A is one of the most plentiful post-transcriptional modifications in mammalian mRNA [14]; many researchers have suggested that m6A modification pattern changes are involved in tumorigenesis, leading to various cancers, e.g., liver [15], lung [16], cervical [17], and pancreatic cancer [18]. Methyltransferase-like 3 (METTL3) was originally identified as responsible for m6A modification and is the pivotal component of the m6A methyltransferase complex [19]. Extensive research has established that METTL3 is involved in cancer progression. METTL3 promotes cancer progression, such as in hepatocellular carcinoma [20], pancreatic cancer [21], lung cancer [22], and acute myeloid leukemia [23]. On the other hand, METTL3 acts as a tumor suppressor in endometrial cancer [24] and triple-negative breast cancer [25]. Therefore, it could have different roles in different cancer types. Recent studies have reported that METTL3 restricts PTC progression through regulating neutrophils infiltration [26, 27]. However, its potential role in PTC and the mechanism by which METTL3 inhibit PTC aggressive phenotypes remains incompletely understood.

Here, we examined the features of STEAP family expression by comprehensively analyzing public databases and tissue microarray. We found that PTC tissue had lower STEAP2 expression than the paired adjoining noncancerous tissue, which was associated with poor prognosis. Notably, gain- and loss-of-function experiments revealed that enforced expression of STEAP2 remarkably inhibited cell proliferation, migration, and invasion in vitro, while silencing STEAP2 yielded the opposite results. In addition, *STEAP2* overexpression inhibited tumorigenesis and lung metastasis in mouse xenografts. Mechanistically, we further identified STEAP2 as a potential direct target regulated by the METTL3-YTHDF1 axis in an m6A-dependent manner. Most importantly, rescue experiments demonstrated that reintroducing STEAP2 markedly abolished the promoting effects on cell proliferation and invasion induced by METTL3 silencing. Overall, we demonstrate that the tumor suppressor protein STEAP2 play a critical roles in PTC progression. Moreover, we provide several new insights into METTL3-mediated *STEAP2* m6A modification, and uncover a novel molecular mechanism underlying PTC.

## Results

### Decreased STEAP2 expression correlates significantly with poor prognosis and metastasis in patients with PTC

**Fig. 1:**
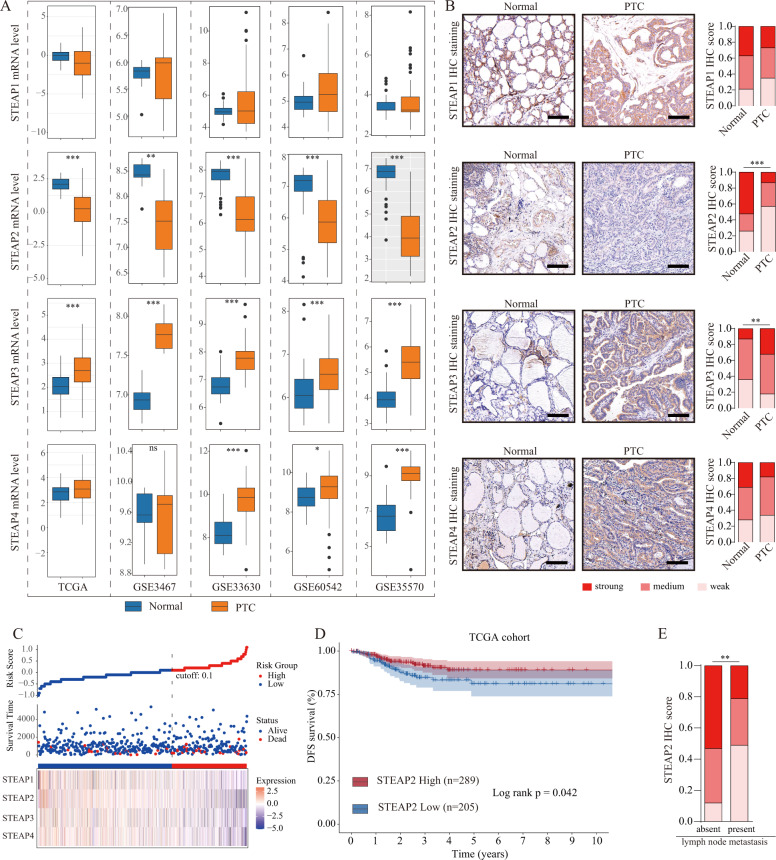
STEAP2 expression is diminished in PTC tissues. **A** Identification of differentially expressed STEAP family members between PTC and normal controls in the TCGA and GEO database (GSE3667, GSE33630, GSE60542, GSE35570). **B** Panel shows representative figures on IHC staining for the 4 STEAP family members (STEAP1/2/3/4) and quantitative data. Scale bar: 200 μm. **C** Risk score distribution, survival status scatter plot, and expression patterns of risk genes in the TCGA PTC cohort. **D** Survival analysis based on STEAP2 expression levels in TCGA PTC cohort (*n* = 494). **E** Distribution of STEAP2 IHC staining scores in PTC tissues according to lymph node metastasis status. *P < 0.05, **P < 0.01, and ***P < 0.001.

To determine the expression status and clinical association between STEAPs in patients with PTC, we first determined the expression profile of STEAP1–4 using public datasets (TCGA and GEO), and further validated with our own clinical PTC tissue cohort through immunohistochemical (IHC) staining. We found STEAP2 and STEAP2 were aberrantly expressed in PTC both at mRNA and protein levels (Fig. 1A, B). Subsequently, we evaluated the clinical significance of STEAPs expression in TCGA PTC cohort, and found that low-level STEAP2 and high-level STEAP1 staining correlated significantly with disease-free survival probabilities (Fig. 1C, D; Supplementary Fig. S1). In addition, STEAP2 was observably lowly expressed in patients with lymph node metastasis (Fig. 1E). In light of the aberrant expression status and good prognostic value of STEAP2 in PTC, we chose STEAP2 for functional investigation.

### STEAP2 ectopic overexpression promotes migration, proliferative, and invasive abilities in vitro

**Fig. 2:**
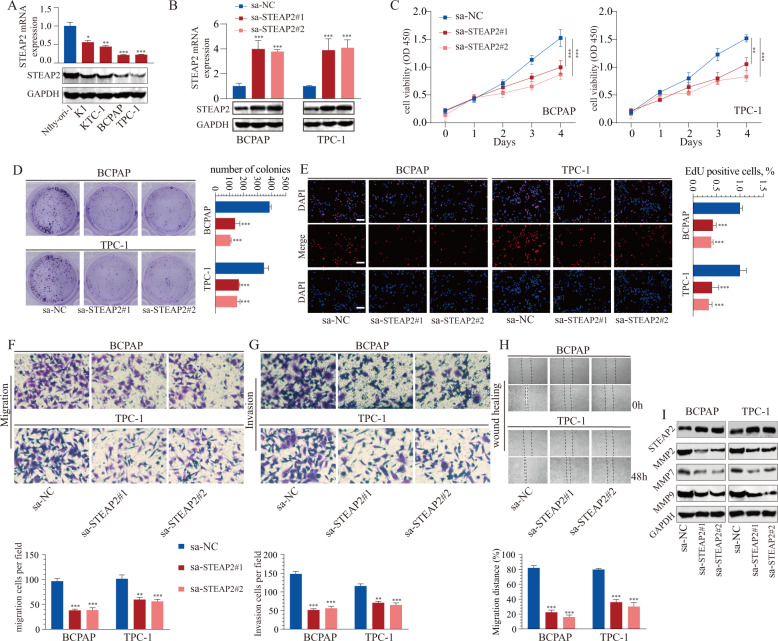
Gain-of-function of STEAP2 inhibits the proliferation and migration of PTC cells. **A** Expression of STEAP2 in normal human thyroid epithelial cell line and PTC cell lines were verified at the mRNA levels (upper panel) and protein levels (bottom panel) via qRT-PCR and western blot, respectively. **B** The activation effect on STEAP2 was detected by qRT-PCR and western blot after transfection of BCPAP and TPC-1 cells with designed saRNAs targeting STEAP2 (sa-STEAP2#1 and sa-STEAP2#2) or negative control (sa-NC). **C**–**E** CCK-8 assay, colony formation assay, and EdU assay were used to determine the viability of BCPAP and TPC-1 cells after STEAP2 activation. Scale bar: 50 μm. **F**–**H** Transwell migration assay, transwell invasion assay, and wound healing assay was performed to evaluate the migration ability of BCPAP and TPC-1 cells after STEAP2 activation. **I** The protein expression of metastasis-mediating proteins (MMP-2, MMP-7, and MMP-9) in PTC cells after indicated transfection was analyzed by western blot. All data are presented as the mean ± standard deviation of three independent experiments. *P < 0.05, **P < 0.01, and ***P < 0.001.

RT-qPCR and western blotting analysis showed that STEAP2 was pervasively lowly expressed in PTC cell lines but expressed at relatively high levels in normal thyroid epithelial cell line (Fig. 2A). Thus, BCPAP and TPC-1 with low expression of STEAP2 were chosen for further gain-of-function studies. BCPAP and TPC-1 cells were transfected with two designed small activating RNA (saRNA) to achieve *STEAP2* overexpression and activation efficiency were validated via RT-qPCR and western blotting (Fig. 2B; Supplementary Fig. S2). After enforced expression of STEAP2, we detected the alterations in cell proliferative ability with CCK-8 assay, EdU testing, and formation of colonies assay. Notably, STEAP2 overexpression markedly inhibited PTC cell proliferative abilities (Fig. 2C–E). In addition, increased TUNEL intensity was observed following STEAP2 overexpression, suggesting enhanced apoptosis (Supplementary Fig. S3). Moreover, transwell and wound healing assay assessment of the role of STEAP2 in PTC cell invasion and migration and showed that STEAP2-activated PTC cells migrated faster as compared to the vector control cells, and invasion activity was increased (Fig. 2F–H). Meanwhile, metastasis-related proteins (MMP2, MMP7, MMP9) were significantly decreased in STEAP2-activated PTC cells (Fig. 2I). More importantly, STEAP2 overexpression has no effect on normal thyroid epithelial cell proliferative abilities (Supplementary Fig. S4). Together, these findings demonstrate that STEAP2 activation diminishes PTC cell proliferation, migration, and invasion in vitro.

### Silencing STEAP2 promotes PTC cell aggressive behavior in vitro

**Fig. 3:**
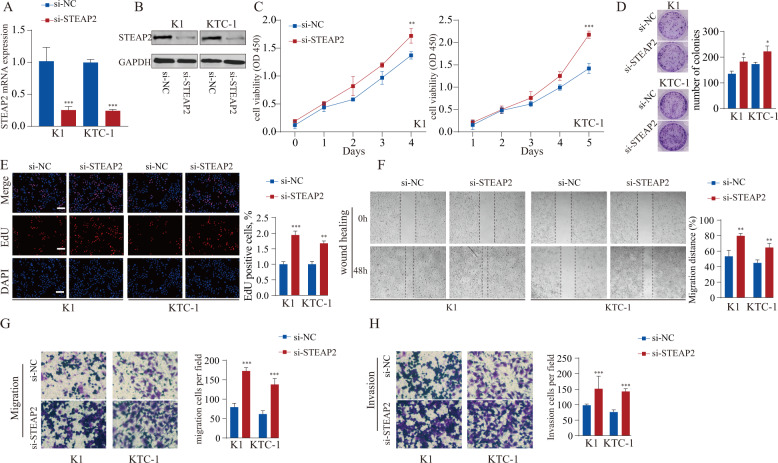
Loss-of-function of STEAP2 inhibits the proliferation and migration of PTC cells. **A-B** The knockdown effect on STEAP2 was detected by RT-qPCR and western blot after transfection of K1 and KTC-1 cells with designed siRNAs targeting STEAP2 (si-STEAP2) or negative control (si-NC). **C**–**E** CCK-8 assay, colony formation assay, and EdU assay were used to determine the viability of BCPAP and TPC-1 cells after STEAP2 silencing. Scale bar: 50 μm. **F**–**H** Wound healing assay, transwell migration assay, and transwell invasion assay were performed to evaluate the migration ability of K1 and KTC-1 cells after STEAP2 activation. All data are presented as the mean ± standard deviation of three independent experiments. *P < 0.05, **P < 0.01, and ***P < 0.001.

In addition to STEAP2 overexpression, loss-of-function experiments were conducted in the STEAP2 relative high-expression PTC cells K1 and KTC-1; knockdown efficiency was confirmed by western blotting and RT-PCR (Fig. 3A, B). The proliferative and clonogenic capacities of the PTC cells were enhanced significantly after STEAP2 knockdown (Fig. 3C, D). In addition, their DNA synthesis rate determined by EdU test was dramatically increased upon *STEAP2* silencing (Fig. 3E). Moreover, migration and invasive abilities of K1 and KTC-1 cells were substantially accelerated following STEAP2 silencing (Fig. 3F–H). Moreover, in order to exclude the possibility of off-target effects, two shRNA targeting STEAP1 with high knockout efficient were employed and consistent results were obtained (Supplementary Fig. S5). Collectively, these observations indicate that STEAP2 could inhibit aggressive tumor phenotypes of PTC cells.

### STEAP2 gain-of-function suppresses tumorigenicity and metastasis in vivo

**Fig. 4:**
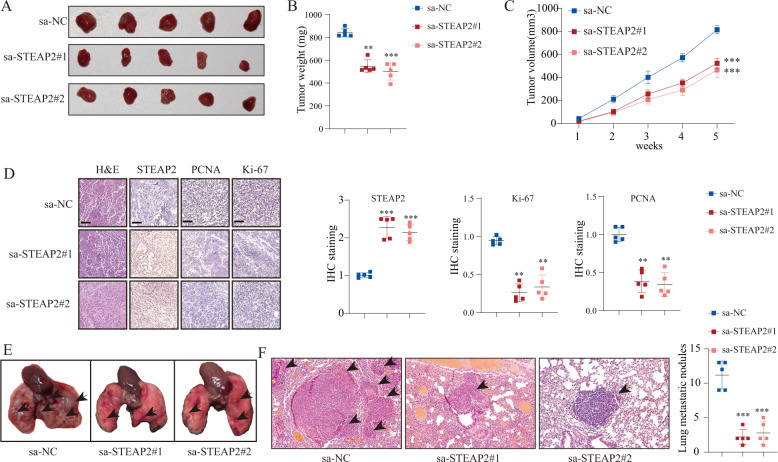
STEAP2 inhibits xenograft growth and lung metastasis of PTC in vivo. For the in vivo tumor metastasis assay, BCPAP cells were injected subcutaneously into the flanks of the mice following multipoint intratumor injection with STEAP2-saRNA or NC-saRNA (**A**). Afterward, tumor weight (**B**) in mice at 31 days after injection and growth curve of xenografts (**C**) were determined. **D** STEAP2, Ki-67, and PCNA expression were detected by IHC in xenografts sections. Scale bar: 100 μm. For the in vivo tumor metastasis assay, BCPAP cells were injected into the tail vein of the mice following tail vein injection with STEAP2-saRNA or NC-saRNA. **E** Representative images of metastatic tumor nodules in the lung of nude mice. **F** Number of metastatic tumor nodules in the lung were compared between nude mice injected with STEAP2-saRNA and NC-saRNA and statistically analyzed. Scale bar: 150 μm. All data are presented as the mean ± standard deviation. *P < 0.05, **P < 0.01, and ***P < 0.001.

We further investigated the effects of STEAP2 reactivation on tumor growth and metastasis in vivo. Tumor growth status in the subcutaneous xenograft mouse model showed that the STEAP2 reactivation group had slower tumor volume growth rate, smaller tumors, and lower final tumor weight than the NC groups (Fig. 4A–C). IHC analysis of STEAP2 expression indicated successful STEAP2 reactivation in vivo (Fig. 4D). In addition, Ki-67 and PCNA staining showed that tumors from the STEAP2 reactivation group had impaired cellular proliferation (Fig. 4D). Consistently, hematoxylin-eosin (HE) staining of resected lung revealed that STEAP2 overexpression significantly decreased lung metastatic colonization (Fig. 4E, F). Conversely, STEAP2 loss-of-function could promote PTC cell tumorigenicity and metastasis in vivo (Supplementary Fig. S6). Taken together, the results show that STEAP2 acts as a novel negative controller of tumorigenicity and metastasis of PTC cells in vivo.

### STEAP2 acts as a tumor suppressor by inhibiting the Hedgehog signaling pathway and EMT

**Fig. 5:**
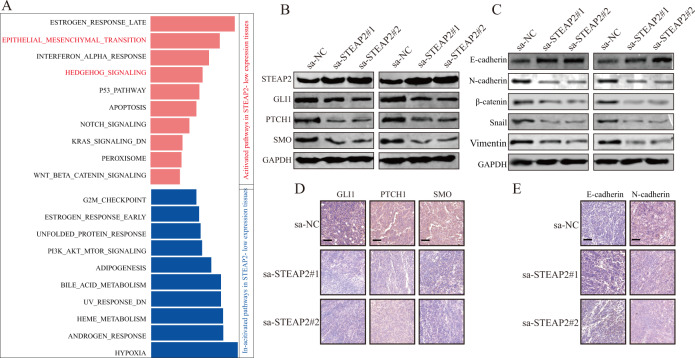
STEAP2 inhibit PTC progression through blocking hedgehog signaling pathway and EMT. **A** GSVA analysis showed that hedgehog signaling pathway and EMT in PTC with STEAP2-low expression compared with those with STEAP2-high expression was identified as the top three activation pathways based on TCGA analysis (red bar: acitivated pathways in STEAP2- low expression tissues; blue bar: In-citivated pathways in STEAP2- low expression tissues). **B** Effect of STEAP2 activation on the expression of Hedgehog signaling pathway-related proteins (SMO, Gli1, and PTCH1) in PTC cells. **C** Representative western blots showing changes in EMT markers (E-cadherin, N-cadherin, snail, vimentin, and β-catenin) in PTC cells after STEAP2 activation. Protein expression levels of Hedgehog signaling pathway-related proteins (Smo, Gli1 and PTCH1) (**D**) and the EMT markers (E-cadherin and N-cadherin) (**E**) in xenograft tumors were determined by IHC. All data are presented as the mean ± standard deviation of three independent experiments. *P < 0.05, **P < 0.01, and ***P < 0.001.

To identify the downstream signaling related to STEAP2, we conducted Gene set variation analysis (GSVA) based on TCGA dataset and found a positive correlation between low STEAP2 expression and abnormal activation of the Hedgehog signaling pathway and epithelial–mesenchymal transition (EMT) (Fig. 5A). It is well known that the Hedgehog signaling pathway is significantly associated with tumor proliferation [28], while EMT plays a crucial role in metastasis [29], indicating that these two signaling pathways may account for the aggressiveness induced by STEAP2 deficiency in PTC. To test this hypothesis, we performed western blotting in vitro and found that STEAP2 ectopic overexpression dramatically suppressed the expression levels of Hedgehog signaling pathway marker proteins including GLI1, PTCH1, and SMO (Fig. 5B). Moreover, decreased SMO phosphorylation level and blocked GLI1 nuclear translocation were observed following STEAP2 activating (Supplementary Fig. S7). In addition, there were decreased mesenchymal molecule marker proteins (N-cadherin, vimentin, Snail, and β-catenin) and increased epithelial molecule marker (E-cadherin) following STEAP2 reactivation (Fig. 5C). Furthermore, we detected the expression of the abovementioned proteins in xenograft tumor tissues via IHC and obtained results consistent with the in vitro findings (Fig. 5D, E). Collectively, these data indicate that STEAP2 may exert its tumor-suppressive function by restraining the Hedgehog signaling pathway and EMT.

### METTL3-regulated m6A modification enhances *STEAP2* mRNA stability in an YTHDF1-dependent manner

**Fig. 6:**
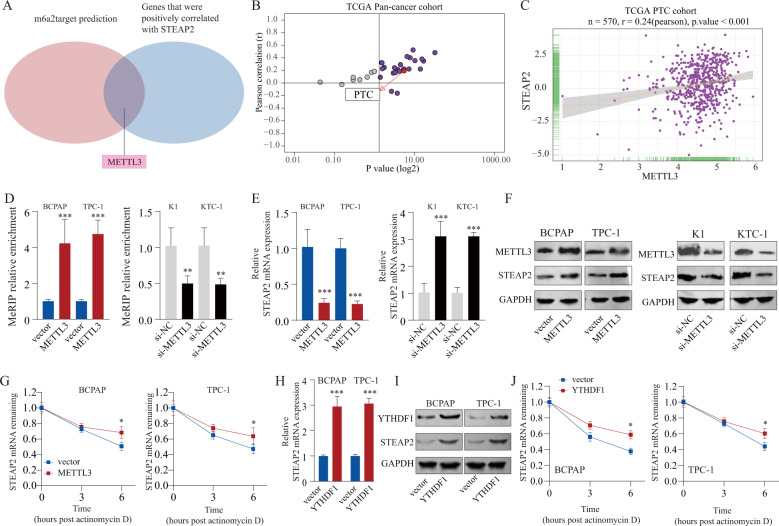
METTL3 enhances STEAP2 mRNA stability via an m6A–YTHDF1-dependent pathway. **A** By retrieving the TCGA dataset and using algorithms (m6a2target), m6a “writer” protein METTL3 were identified to be able to potentially interact with STEAP2 mRNA. Pearson correlation analysis between STEAP2 and METTL3 mRNA levels in Pan-cancer cohort (**B**) and PTC cohort (**C**) retrieved from TCGA. **D** MeRIP-qPCR analysis was employed to demonstrate METTL3-mediated STEAP2 m6A modifications. The m6A modification of STEAP2 was increased on upregulation of METTL3, while it was depleted on METTL3 silencing. The expression levels of STEAP2 in METTL3-overexpressing and METTL3 knockdown PTC cells were detected by qRT-PCR (**E**) and western blot (**F**). **G** STEAP2 mRNA level in METTL3-overexpressing or METTL3-knockdown PTC cells treated with actinomycin D at the indicated time points were detected by qRT-PCR. The expression levels of STEAP2 in YTHDF1-overexpressing PTC cells were detected by qRT-PCR (**H**) and western blot (**I**). **J** STEAP2 mRNA level in TYTHDF1-overexpressing PTC cells treated with actinomycin D at the indicated time points were detected by qRT-PCR. All data are presented as the mean ± standard deviation of three independent experiments. *P < 0.05, **P < 0.01, and ***P < 0.001.

Increasing studies have shown that m6A RNA modification is an emerging regulatory mechanism for gene expression and plays vital roles in tumorigenesis [30]. Hence, we explored whether m6A modification was involved in STEAP2 inactivation. Online prediction tool (http://m6a2target.canceromics.org [31]) showed that METTL3, a m6A “writer” protein, was among the potential m6A functional candidates regulating STEAP2 expression, while persistently positive relationship between METTL3 and STEAP2 expression was observed in most cancers, including PTC (Fig. 6A–C). Previous study reported that METTL3 is downregulated in PTC tissues and plays a tumor-suppressor role [26]. To confirm whether *STEAP2* mRNA undergoes METTL3-mediated m6A modification, we performed methylated RNA immunoprecipitation quantitative PCR (MeRIP-PCR) and results indicated that the m6A abundance of *STEAP2* mRNA was notably decreased upon METTL3 silencing, while it was increased following METTL3 upregulation (Fig. 6D). Moreover, STEAP2 expression was significantly enhanced after METTL3 reintroduction, whereas it was decreased with METTL3 knockdown in PTC cells at both mRNA and protein levels (Fig. 6E, F; Supplementary Fig. S8). As expect, *STEAP2* mRNA stability was enhanced upon METTL3 overexpression, and was decreased in METTL3-silenced PTC cells (Fig. 6G; Supplementary Fig. S9). More importantly, the effect of catalytic mutant METTL3 construct on STEAP2 was assessed. As expected, STEAP2 expression in METTL3-Mutant-transfected cells was no different from controls group, indicating that METTL3 regulates STEAP2 via distinct mechanisms which depends on the catalytic activity of METTL3 (Supplementary Fig. S10).

The current consensus indicates that m6A modification exercises its biological functions mainly by recruiting m6A “reader” proteins [32]. Among the identified m6A reader proteins, YTHDF1 promotes m6A-modified mRNA protein translation [33]. Intriguingly, significant increased STEAP2 expression was observed in PTC cells following YTHDF1 reintroduction, while not YTHDF2 (Fig. 6H, I; Supplementary Fig. S11). In addition, YTHDF1 overexpression could enhance *STEAP2* mRNA stability, while rescue experiments showed that YTHDF1 silencing remarkable counteracted the positive effects on STEAP2 expression induced by METTL3 overexpression (Fig. 6J; Supplementary Fig. S12). In summary, these findings suggest that METTL3-mediated m6A modification enhance *STEAP2* mRNA stability in an YTHDF1-dependent manner.

### Silencing STEAP2 reverses the tumor inhibitory effects of METTL3

**Fig. 7:**
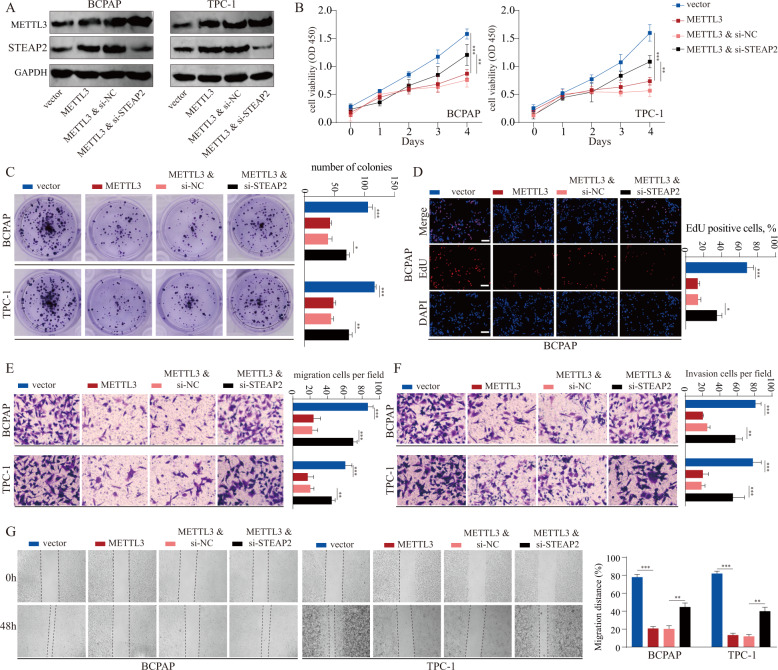
METTL3 accelerates PTC malignant progression by upregulating STEAP2. **A** BCPAP and TPC-1 cell lines were transfected with METTL3-overexpression (METTL3) plasmids and siRNA targeting STEAP2 (si-STEAP2) as indicated. The expression levels of METTL3 and STEAP2 were quantified by western blot assay. The effects of METTL3 and STEAP2 on proliferative ability of PTC cells were measured by CCK-8 assay (**B**), colony formation assay (**C**), and EdU assay (**D**). The effects of METTL3 and STEAP2 on invasive ability of PTC cells were determined by transwell migration assay (**E**), transwell invasion assay (**F**), and wound healing assay (**G**). All data are presented as the mean ± standard deviation of three independent experiments. *P < 0.05, **P < 0.01, and ***P < 0.001.

We further verified whether STEAP2 inactivation was involved in the tumor inhibitory activity of METTL3 in PTC. We enforced METTL3 expression in BCPAP and TPC-1 cells with STEAP2 silencing by siRNA (Fig. 7A). Rescue experiments showed that the transiently transfected STEAP2 siRNA could partially rescue the negative effects on cell proliferation induced by METTL3 (Fig. 7B–D; Supplementary Fig. S13A). In addition, TUNEL assay results showed that STEAP2 knockdown could rescue the increased apoptosis rate in PTC cells transfected with METTL3 overexpression plasmid (Supplementary Fig. S13B). Moreover, STEAP2 silencing partially abrogated the migration and invasion inhibitory effects caused by METTL3 overexpression in PTC cells (Fig. 7E–G).

**Fig. 8:**
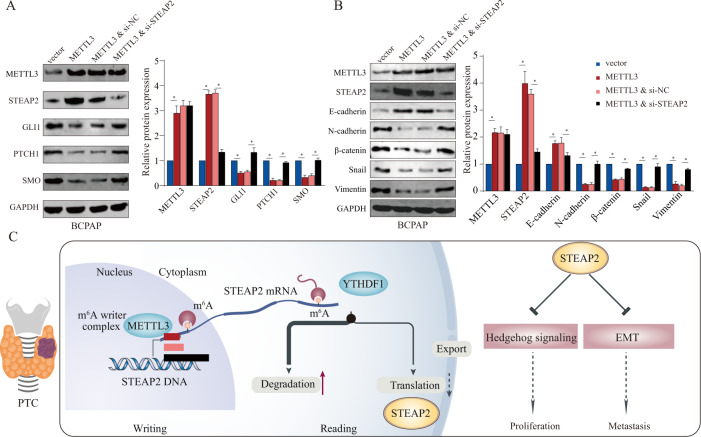
METTL3/STEAP2 m6A axis regulates hedgehog signaling pathway and EMT in PTC. BCPAP PTC cell lines were transfected with METTL3-overexpression (METTL3) plasmids and siRNA targeting STEAP2 (si-STEAP2) as indicated. **A**, **B** Effect of METTL3/STEAP2 axis on the expression of Hedgehog signaling pathway-related proteins (Smo, Gli1, and PTCH1) and EMT markers (E-cadherin, N-cadherin, snail, vimentin, and β-catenin) in PTC cells were quantified by western blot assay. **C** The graphic illustration of METTL3-mediated m6A modification modulating malignant phenotype of PTC through enhancing STEAP2 expression and then restraining Hedgehog signaling pathway and EMT. All data are presented as the mean ± standard deviation of three independent experiments. *P < 0.05, **P < 0.01, and ***P < 0.001.

Furthermore, we evaluated the effect of the METTL3–STEAP2 axis on the Hedgehog signaling pathway and EMT. The results suggested that METTL3 upregulation inhibited Hedgehog signaling pathway and EMT activation, while STEAP2 knockdown partially restored the inhibitory effects on the Hedgehog signaling pathway and EMT in METTL3 overexpression PTC cells (Fig. 8A, B). Therefore, our data suggest that METTL3 suppresses the PTC malignant process by enhancing STEAP2 expression and consequently restraining the Hedgehog signaling pathway and EMT.

## Discussion

STEAP family members are extensively expressed in noncancerous human tissues and were initially identified as important metalloreductases participating mineral absorption, ferroptosis, and TP53-regulated transcription of cell death genes [6]. Emerging evidence has shown that STEAPs are frequently aberrantly expressed in a variety of cancer types [4]. Functionally, STEAPs play both tumor-suppressive or -promoter roles in cancer progression [4]. Nevertheless, their function in PTC is still undefined.

Here, the expression status and prognostic significance of all STEAP family members underwent systematic and comprehensive analysis. We identified that PTC tissues had obviously downregulated STEAP2, and reduced STEAP2 expression correlated worse prognosis. Functionally loss-of-function assays demonstrated the silencing STEAP2 promoted PTC cell invasion and proliferation in vitro. Likewise, STEAP2 knockdown enhanced lung metastasis and tumor growth in vivo. Conversely, STEAP2 gain-of-function had the opposite effects. These results suggest that STEAP2 may have an anti-oncogenic role in PTC progression. In accordance with our findings, downregulated STEAP2 has been observed in breast cancer and glioblastoma, while STEAP2 downregulation promotes breast cancer cell proliferation and invasion [34–36]. On the contrary, it has been reported STEAP2 is overexpressed in other human cancers, e.g., bladder, pancreatic, ovarian, cervical, colon cancer, and Ewing sarcoma [4]. Burnell et al. reported that *STEAP2* knockdown decreased the potential for prostate cancer cells to invade [37]. We assume that STEAP2 may have opposite functions due to the heterogeneity of different cancers.

Previous studies demonstrated that STEAP2 inhibited EMT and suppressed the PI3K–AKT–mTOR signaling pathway in breast cancer [35]. Intriguingly, bioinformatics analysis indicated that the Hedgehog signaling pathway and EMT were the most significantly enriched pathways in STEAP2-low expression PTC tissues. The Hedgehog signaling pathway is highly conserved, and previous studies have demonstrated that its aberrant activation has a vital function in PTC progression and development [38]. Meanwhile, EMT allows epithelial cells obtain the invasive and motile abilities characteristic of mesenchymal cells, a vital development in cancer metastasis [29]. Here, we uncovered a new mechanism where STEAP2 blocked Hedgehog signaling pathway and inhibit EMT activation. However, more studies should be performed to clarify the underlying mechanism for this crosstalk.

To date, there has been no report on the regulatory mechanism of aberrant STEAP2 expression. m6A modification is a widespread internal mRNA modification that regulates the result of gene expression [30]. In the present study, we verified that the METTL3-initiated m6A methylation increases *STEAP2* mRNA stability and promotes its translation in a YTHDF1-dependent pathway. METTL3 is the master component of the writer complex and plays key roles in regulating gene expression [32]. METTL3 was reported to display a dual role as both an oncogene and a tumor suppressor [39]. Previous publications have reported that METTL3 is downregulated in PTC and functions as a suppressor by regulating neutrophil infiltration, which was in line with our observations [26]. In the present study, rescue experiments revealed that silencing STEAP2 partially rescued the tumor-suppressive phenotype induced by METTL3 overexpression. Notably, upregulating METTL3 blocked the Hedgehog signaling pathway and EMT activation, and disrupting *STEAP2* partially abrogated these inhibitory effects. Taken together, these results indicate that METTL3 induces *STEAP2* translation in an m6A-dependent manner, subsequently leading to inactivation of the Hedgehog signaling pathway and EMT, and restrains aggressive tumor phenotypes (Fig. 8C).

## Conclusion

To our knowledge, this represents the first systematic analysis of the role of STEAP2 in PTC. STEAP2 was characterized as a potential tumor suppressor and could inhibit tumor proliferation and metastasis in PTC. We also demonstrate that METTL3 epigenetically enhances STEAP2 expression via an m6A–YTHDF1-dependent mechanism. The discovery of the METTL3–STEAP2 axis and its impact on PTC progression will aid further exploration of efficient therapeutic strategies against aggressive PTC.

## Methods

### Clinical tissue specimens

Twenty PTC patients having went through resection at the Sun Yat-Sen Memorial Hospital were recruited in this cohort study. The entire procedures were subjected to the supervision of the Ethics Review Committee of Sun Yat-Sen Memorial Hospital, and rigorously remained in conformance to the Declaration of Helsinki. All involved subjects submitted informed written consent before tissue sampling.

### Analysis of public databases

The raw expression data for PTC samples were gained from the Cancer Genome Atlas (TCGA) and GEO database. The individual datasets (GSE3467, GSE33630, GSE35570, GSE60542) were analyzed in this study.

### Immunohistochemistry analysis

The slides were subjected to 30 min of H_2_O_2_ solution (0.3%) treatment to inhibit the activity of endogenous peroxidase. Permeabilization was conducted by treating tissues with 0.5% Triton X-100 for 30 min, followed by incubation of tissues with citrate buffer at working strength to identify the relevant antigen. Following three times of rinsing with PBS, 5% normal goat serum was used to block the slides. Samples were subsequently subjected to cross-reaction with STEAP1 antibody (Proteintech: 20199-1-AP, China), STEAP2 (Proteintech: 20201-1-AP, China), STEAP3 (Proteintech: 17186-1-AP, China), STEAP4 (Proteintech: 11944-1-AP, China), SMO (Proteintech: 20787-1-AP, China), GLI1 (Abcam: ab134906, USA), PTCH1 (Abcam: ab53715, USA), Ki-67 (Proteintech: 27309-1-AP, China), PCNA (Proteintech: 10205-2-AP, China), E-Cadherin (Proteintech: 20874-1-AP, China), N-Cadherin (Proteintech: 22018-1-AP, China). Dilution of antibodies was performed at a ratio of 1:100, followed by two hours of incubation with the sample at ambient temperature. The slides were then subjected to 30 min of incubation with a secondary horseradish peroxidase (HRP)-conjugated anti-rabbit IgG (H + L). Besides, counterstaining of nuclei was carried out with DAPI.

### saRNA activation, RNA interference, and transfection

saRNA was designed as specified in previous reports [40]. Three double-stranded RNA pairs, containing 21 nucleotides each, were designed as complementary to the sequence of STEAP3 promoter. The saRNA sequences were listed in Supplementary Table S1. Synthesis of saRNA was performed in GeneBio, China. The STEAP2-siRNA (si-STEAP2), METTL3-siRNA (si-STEAP2), and negative control (si-NC) siRNAs were procured from RiboBio, China. The pcDNA3.1-METTL3 and empty vectors were procured from Sangon Biotech, China. Transfection with plasmids or siRNAs was conducted on Lipofectamine 3000 (Invitrogen, USA) as per the official protocol.

### CCK-8 assay, EdU analysis, and colony formation assay

Cell viability was evaluated using CCK-8 as well as with Ethynyl deoxyuridine (EdU) assay and colony formation assay. Spreading of PTC cells was performed in 96-well plates with the density at 1 × 10^4^ cells/ml. After 0, 24, 48, and 72 h of incubation, respectively, each well was supplemented with CCK-8 solution (Djingo, Japan) and the OD at 450 nm was detected with a microplate reader. EdU assay was conducted as specified by the vendor (RiboBio, China). Treated cells were detected under a fluorescence photo-microscopy, and measured by counting the numbers in at least 6 random fields. For colony formation assay, cells were incubated in a complete medium on plates for 14 days. The medium was replaced at an interval of 3 days. Cell colonies were subjected to fixation with paraformaldehyde, and subsequently to 20 min of staining with crystal violet (0.1%). Visually available colonies were quantified.

### RT-qPCR

TRIzol reagent (Invitrogen) was used to extract Total RNA. Takara kit (Dalian, China) was used for performing reverse transcription reactions at 42 °C. RT-PCR analyses were conducted using SYBR Green supermix (Bio-Rad Laboratories, U.S.) according to a two-step procedure. GAPDH was used for normalizing gene expressions. ΔΔCt or 2-ΔΔCt method was applied for data analysis. Synthesis of primers was conducted in Sangon Biotech (Shanghai, China) with the sequences as follows: STEAP2 F: 5′-GGTCACTGTAGGTGTGATTGG-3′, R: 5′-ACCACATGATAGCCGCATCTAA-3′; GAPDH R: 5′-CCAGGTGGTCTCCTCTGA-3′, GADPH F: 5′-GCTGTAGCCAAATCGTTGT-3′.

### Western blot

Extraction of total proteins was performed with RIPA lysis buffer containing protease inhibitors. Proteins were detected using a BCA protein assay kit (Pierce, China) prior to being separated by SDS-PAGE and migrated to PVDF membranes (Millipore, Germany). The membranes were then subjected to incubation with respective primary antibodies against STEAP2 (Proteintech, 20201-1-AP), METTL3 (Proteintech, 15073-1-AP), YTHDF1 (Proteintech, 17479-1-AP), SMO (Proteintech, 20787-1-AP), GLI1 (Abcam, ab134906), PTCH1 (Abcam, ab53715), E-Cadherin (Proteintech, 20874-1-AP), N-Cadherin (Proteintech, 22018-1-AP), MMP2 (Proteintech, 10373-2-AP), MMP7 (Proteintech, 10374-2-AP), MMP9 (Proteintech, 10375-2-AP), SNAIL (Proteintech, 13099-1-AP), β-catenin (Proteintech, 51067-2-AP), Vimentin (Proteintech, 10366-1-AP). The signal was examined with a LI-COR Odyssey Imaging System (Lincoln, NE, USA) as specified by the vendor.

### Transwell migration and invasion assay

Transwell assay was performed to determine cell migration and invasion. Cells were seeded in 24-well Transwell inserting chambers (BD Biosciences, USA) (2% Matrigel was used to pre-coat inserting chambers for invasion assay) that contained serum-free medium. FBS (20%) was added to the lower-chamber medium as a source of chemo-attractant. After 2 days, the cells having not migrated through the chamber membrane were removed, and those having passed through the membrane were subjected to fixation and staining with crystal violet. They were subsequently detected and photographed using a microscope.

### In vivo tumor xenograft model and lung metastasis model

The animal experiment permission was granted by the Animal Care Committee of the Sun Yat-Sen Memorial Hospital. Female BALB/c nude mice (aged between 6 and 8 weeks) were procured from the Beijing Vital River Laboratory Animal Technology Company, and reared in conditions deprived of specific pathogens. BCPAP cells (5 × 10^6^) were introduced into the mice by means of subcutaneous injection through the flank area. STEAP2-saRNA or NC-saRNA (*n* = 6 for each group) was given by intratumoral multipoint injection at an interval of 3 days (5 injections in total) using an in vivo transfection reagent (Entranster™-in vivo, Engreen, China) as per the vendor-provided protocol. Tumor volume (*V*) was monitored and calculated as follows: *V* = (*L* × *W*^2^)/2. For the in vivo tumor metastasis assay, BCPAP cells (5 × 10^6^ cells) were administrated into mice through the tail vein. STEAP2-saRNA or NC-saRNA (*n* = 6 for each group) was given via tail vein injection at an interval of 3 days (8 injections in total). Lung micrometastases were counted via morphological observation of H&E-stained sections.

### RNA total m6A quantification

GenElute™ mRNA Miniprep Kit (Sigma-Aldrich, Germany) was used to extract total RNA and remove polyadenylated mRNA by following the vendor-provided instructions. Then, the total m6A level was determined with deEpiQuik™ m6A RNA Methylation Quantification Kit (Colorimetric) (Epigentek, USA) in treated cells as per the specific protocol. The m6A content was quantified based on the absorbance at 450 nm.

### MeRIP-qPCR

Magna MeRIP™ m6A Kit (Millipore, Germany) was used for MeRIP assay to identify the m6A modification of specific transcripts. Briefly, a total of 150 μg RNA was extracted from pretreated cells, and reduced into fragments of 100 or fewer nucleotides. Immunoprecipitation of RNA samples was performed with magnetic beads pre-coated with 10 μg anti-m6A antibody (Millipore) or anti-mouse IgG (Millipore). Normalization of m6A enrichment was performed relative to inputs.

### Statistical analysis

All experiments were performed in 3 repeated runs at a minimum. All statistical data were processed on GraphPad Prism 7.0 (GraphPad, USA), and shown as mean ± SD. Nonpaired Student’s *t*-test or nonparametric Mann–Whitney test was used for two-group comparison. One-way or two-way ANOVA, along with the Bonferroni post hoc test, was adopted in comparisons between more than two groups. The linear relationship between METTL3 and STEAP2 expression levels was measured by Pearson correlation coefficient. The survival probability was calculated using the Kaplan–Meier approach and compared with the log-rank test. All statistical analysis involved two-tailed tests. Statistical significance was denoted by *P* < 0.05.

## Supplementary information

SUPPLEMENTAL MATERIAL

Supplementary table S1

## Data Availability

The datasets used and/or analyzed during the current study are available from the corresponding author on reasonable request.
