# Supplementary material for: METTL3-mediated m6A modification of STEAP2 mRNA inhibits papillary thyroid cancer progress by blocking the Hedgehog signaling pathway and epithelial-to-mesenchymal transition
Source: Cell Death Dis. 2022 Apr 18;13(4):358. doi: 10.1038/s41419-022-04817-6 (PMC9016063; doi:10.1038/s41419-022-04817-6)
Supplement: Supplementary file 1 — SUPPLEMENTAL MATERIAL [file 41419_2022_4817_MOESM1_ESM.pdf]

Supplementary Figure S1

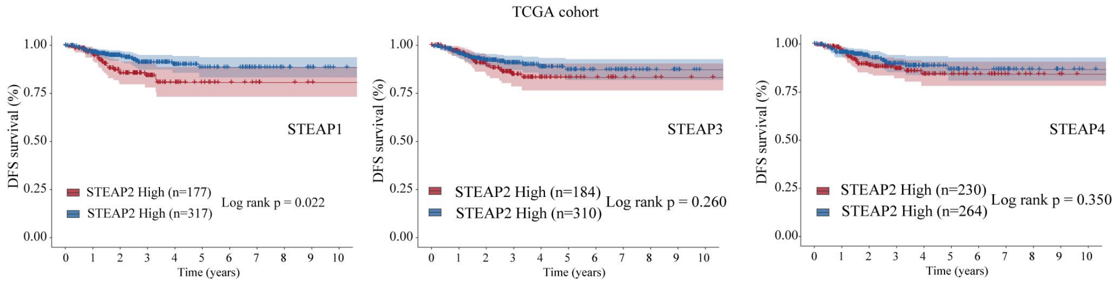

Supplementary Figure S2

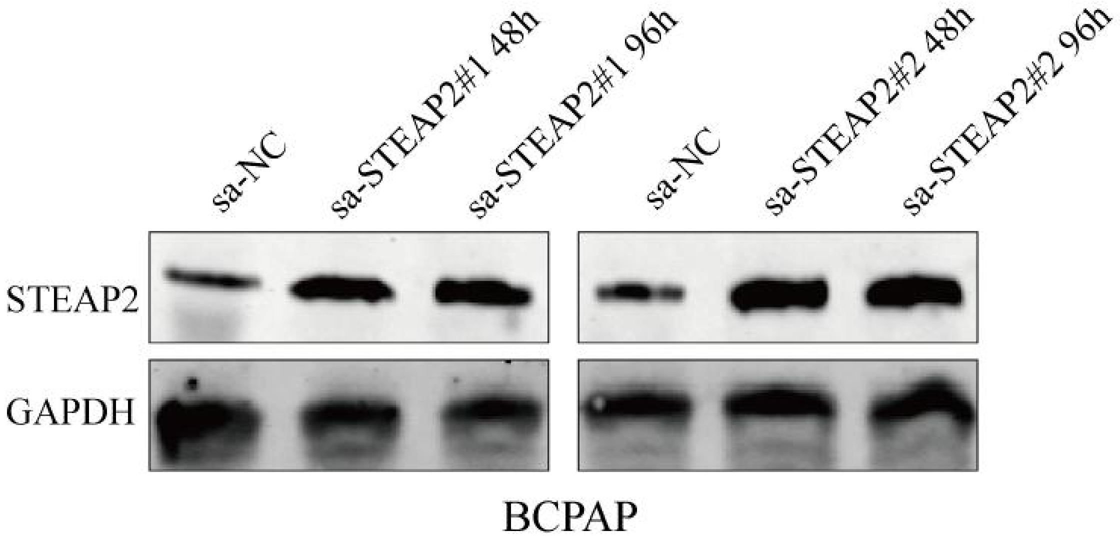

Supplementary Figure S3

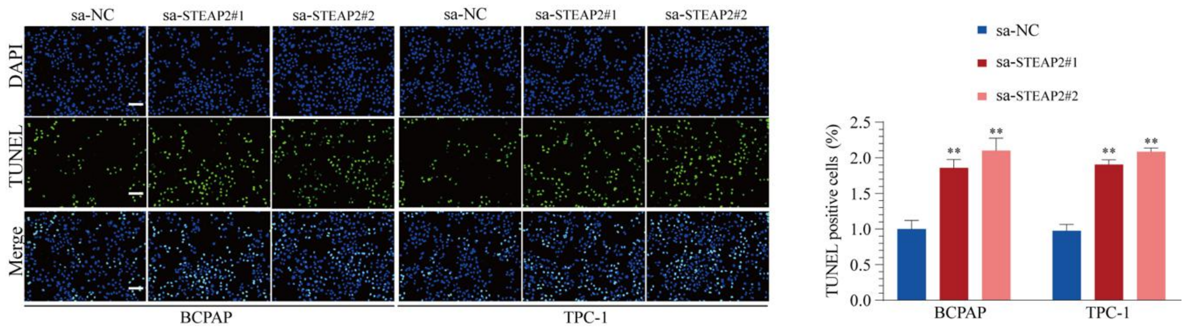

Supplementary Figure S4

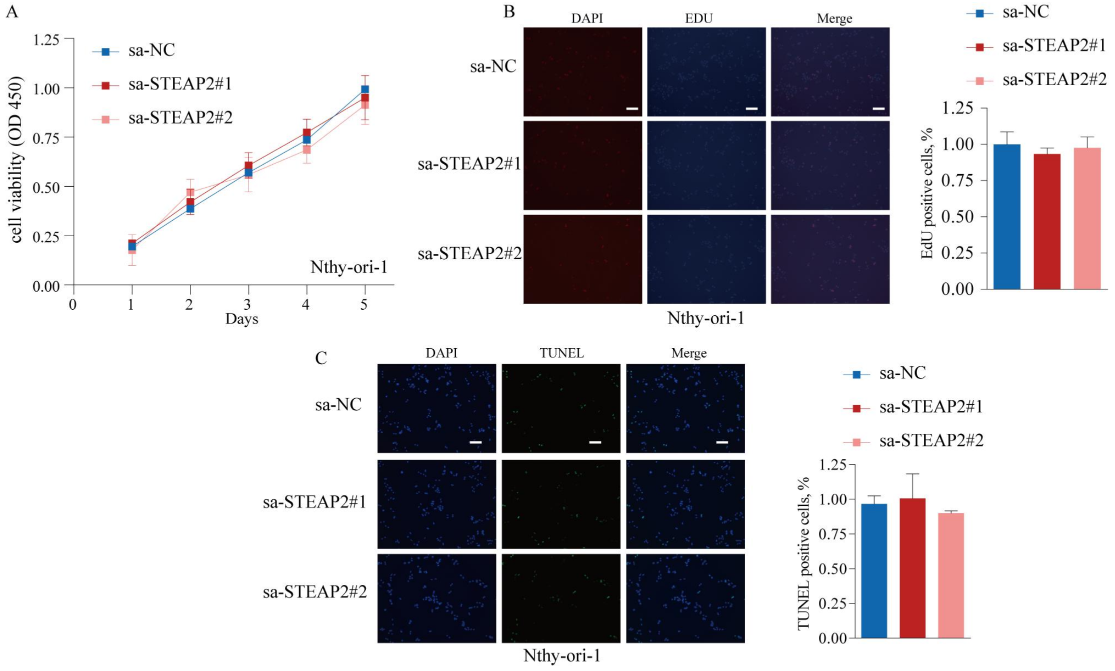

Supplementary Figure S5

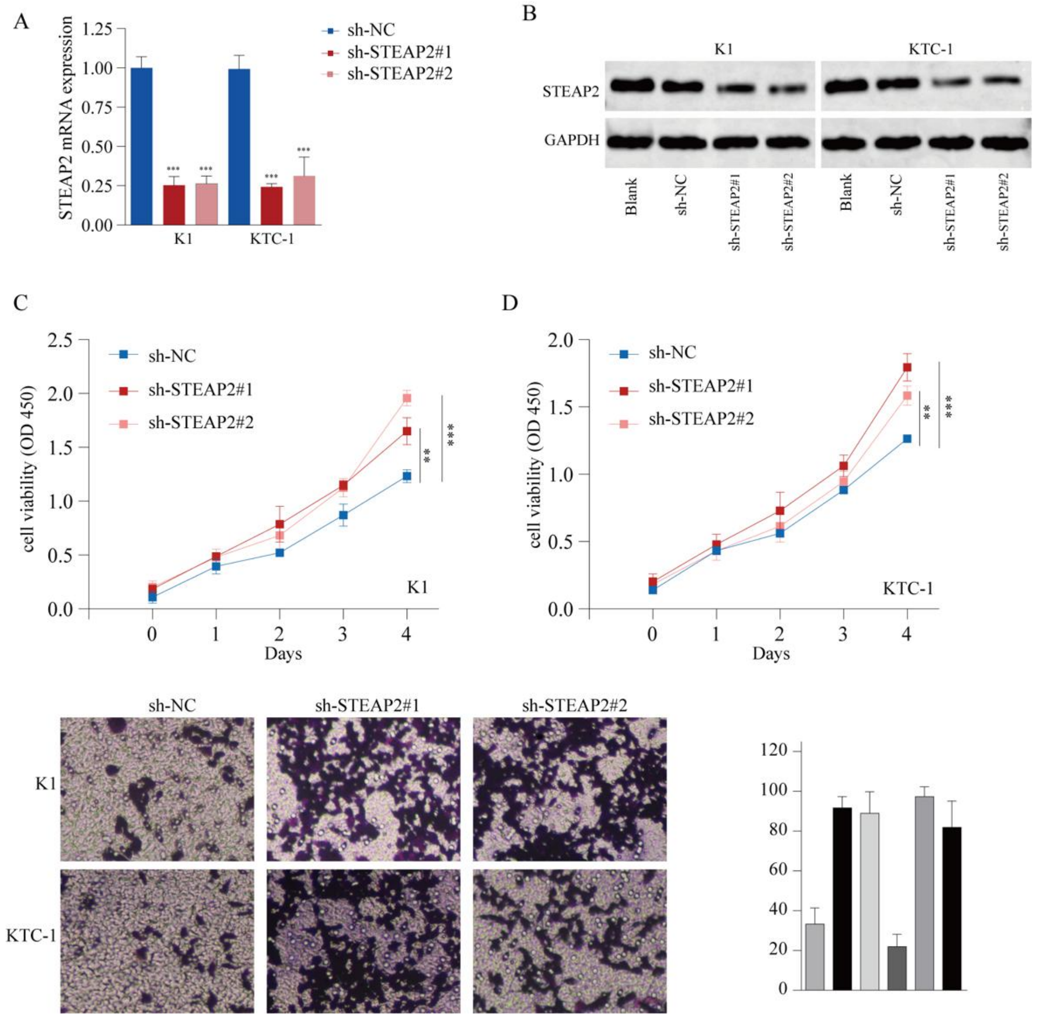

Supplementary Figure S6

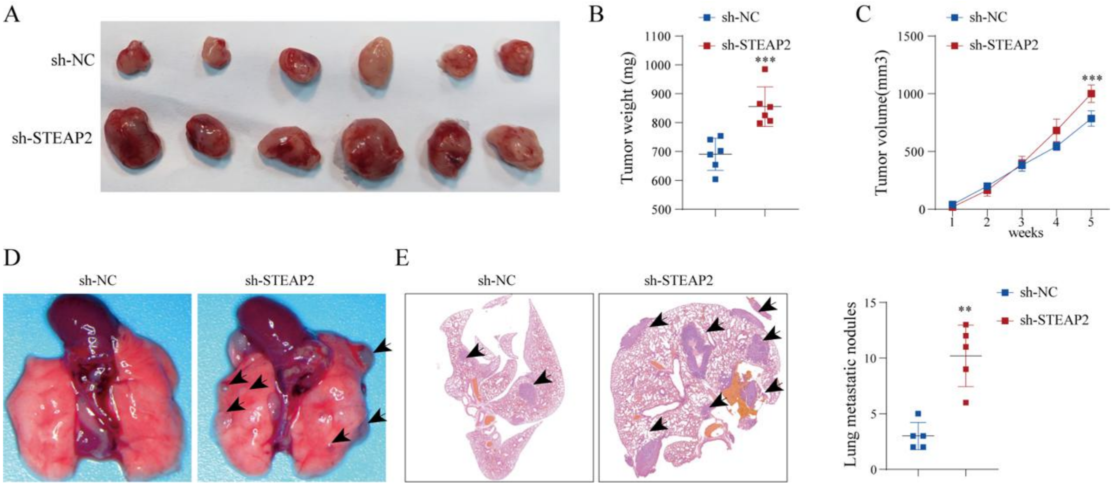

Supplementary Figure S7

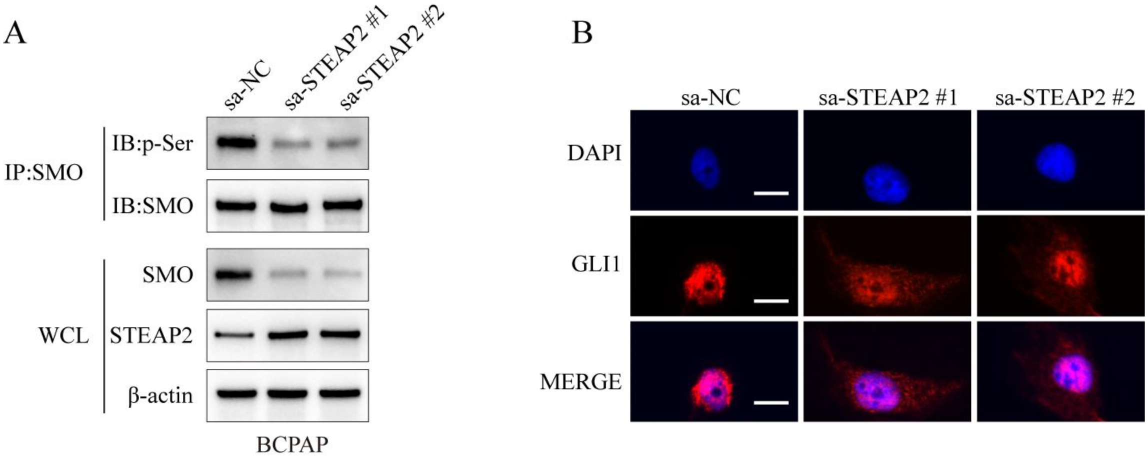

Supplementary Figure S8

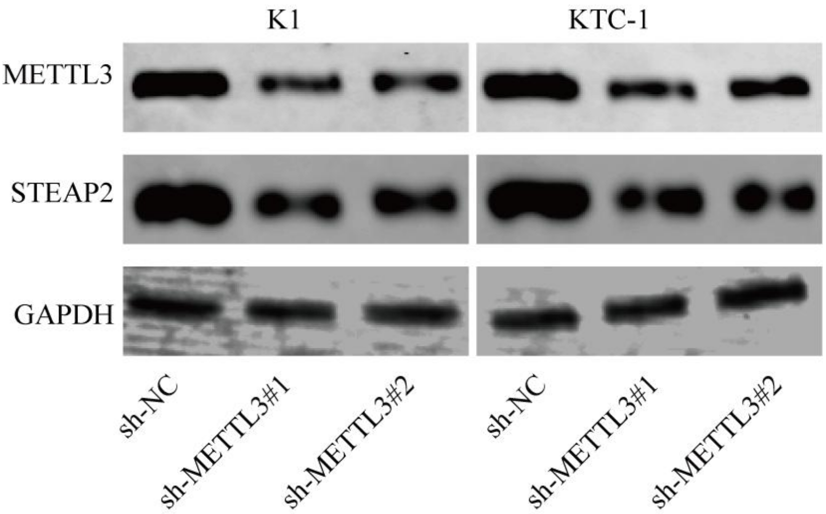

Supplementary Figure S9

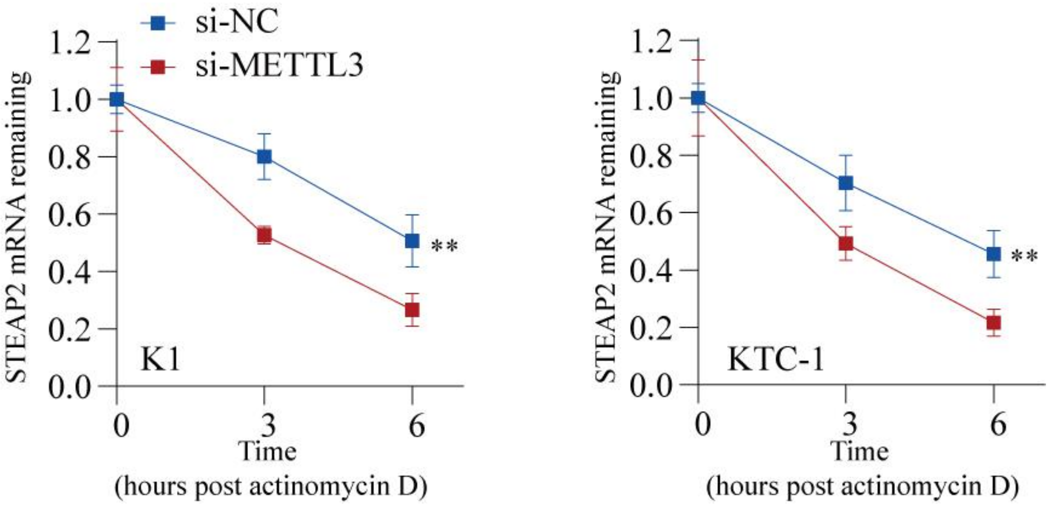

Supplementary Figure S10

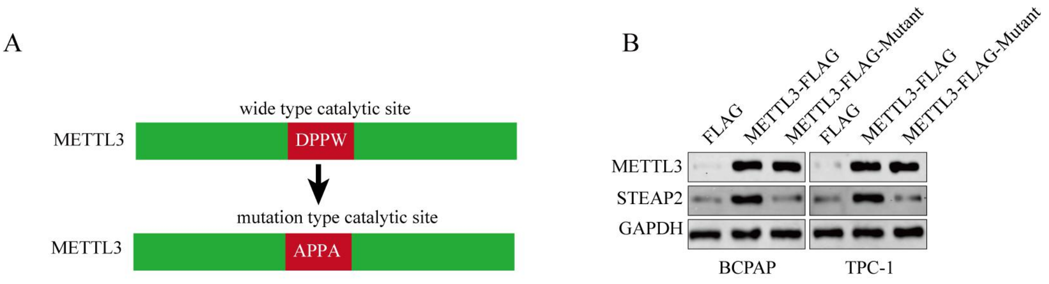

Supplementary Figure S11

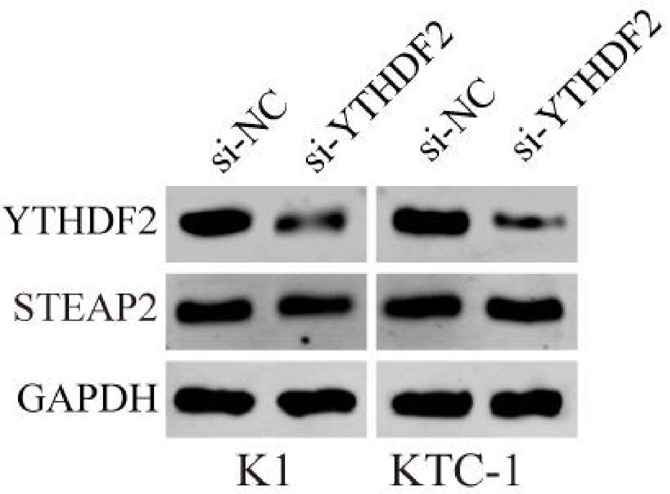

Supplementary Figure S12

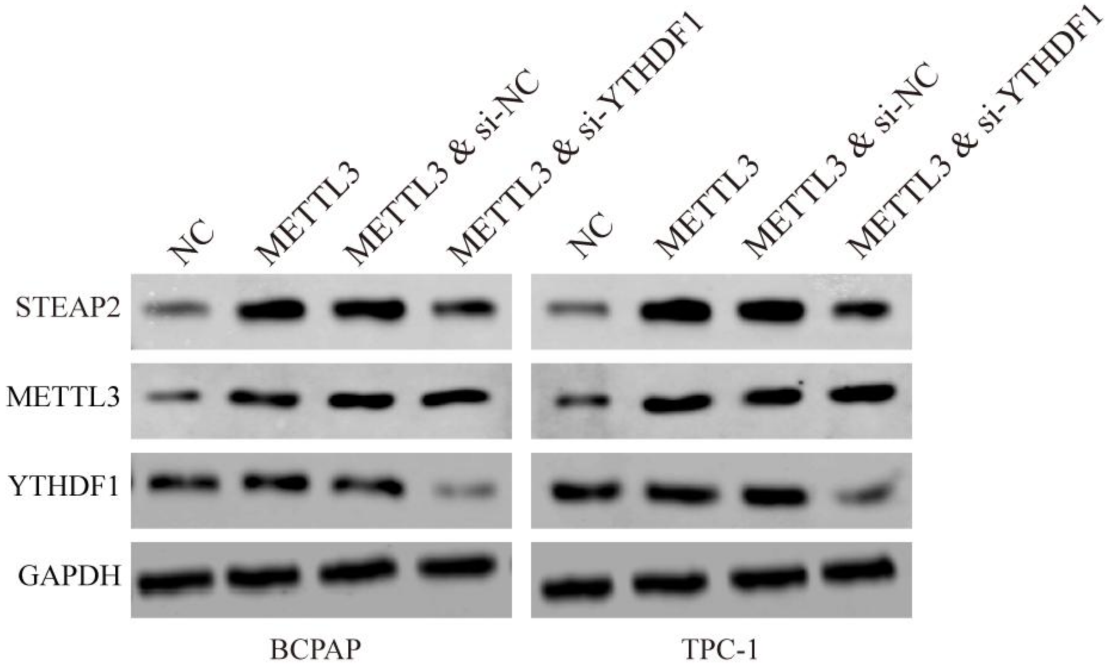

Supplementary Figure S13

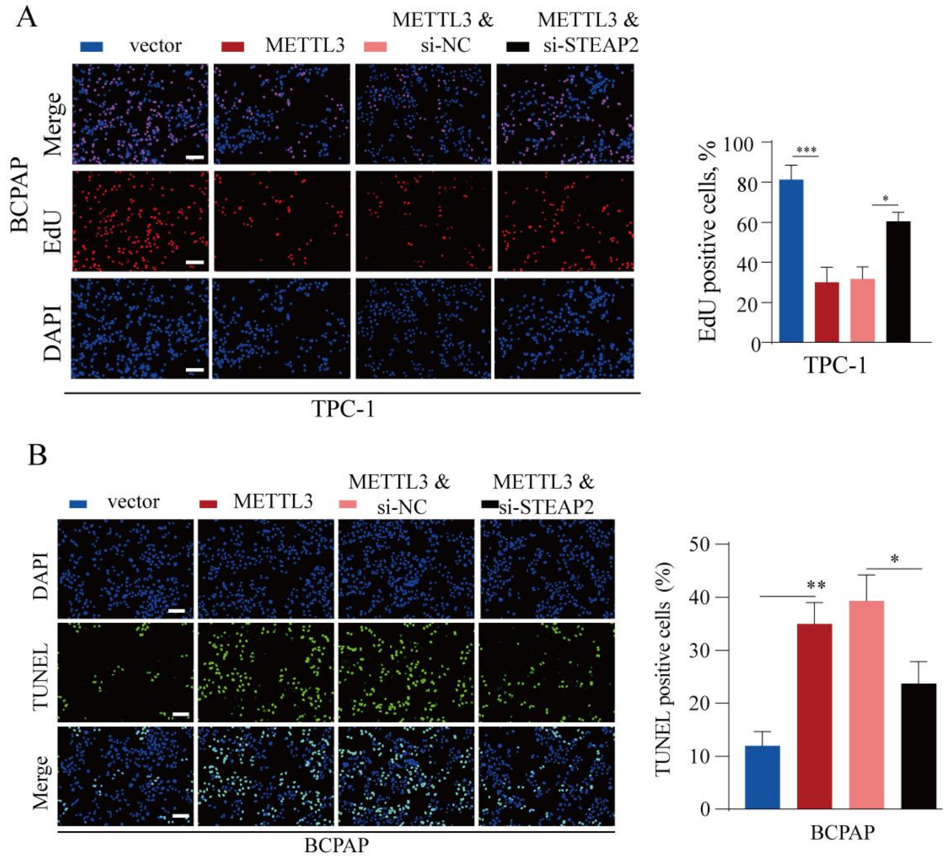

Raw western blot membranes

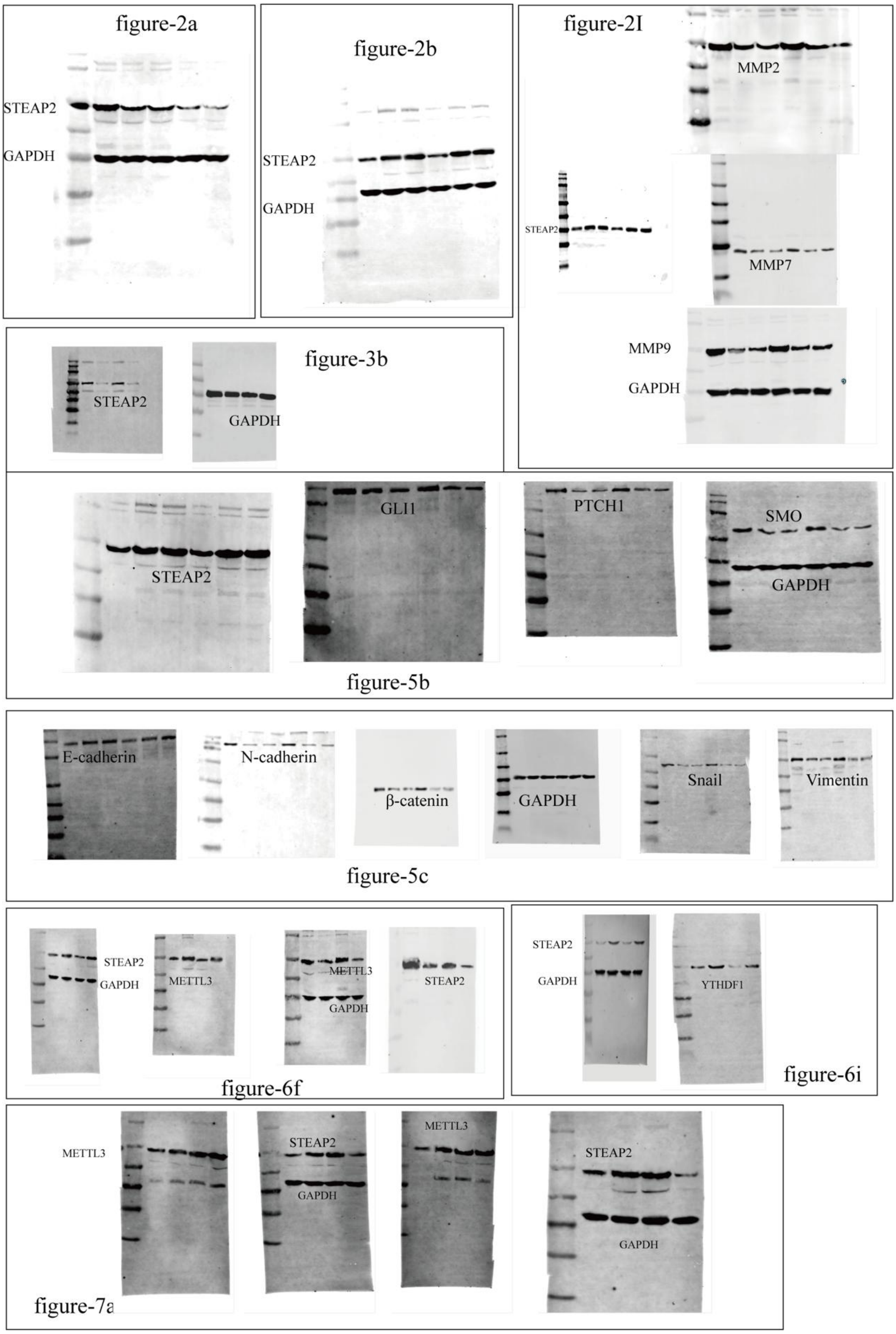

Supplementary Figure legends

Supplementary Figure S1. Survival analysis based on STEAP1, STEAP3 and STEAP4 expression levels in TCGA PTC cohort (n=494).

Supplementary Figure S2. The activation effect on STEAP2 was detected by western blot after transfection of BCPAP and TPC-1 cells with designed saRNAs targeting STEAP2 (sa-STEAP2#1 and sa-STEAP2#2) or negative control (sa-NC) at 48 hours and 96 hours.

Supplementary Fig. S3. TUNEL assay were used to determine the apoptosis rate of BCPAP and TPC-1 cells after STEAP2 activation. All data are presented as the mean ± standard deviation of three independent experiments. Scale bar: 50 μm. *P < 0.05, **P < 0.01, and ***P < 0.001.

Supplementary Fig. S4. CCK-8 assay (a), EdU assay (b) and TUNEL assay (c) were used to determine the viability of normal thyroid epithelial cell Nthy-ori-1 after STEAP2 activation. Scale bar: 50 μm.

Supplementary Fig. S5. The knockdown efficiency on STEAP2 was detected by qRT-PCR (a) and western blot (b) after transfection of K1 and KTC-1 cells with designed shRNAs targeting STEAP2 (sh-STEAP2#1 and sh-STEAP2#2) or negative control (sh-NC). (c, d) CCK-8 assay were used to determine the viability of K1 and KTC-1 cells after STEAP2 silencing. (e, g) Transwell migration assay and invasion assay was performed to evaluate the migration ability of K1 and KTC-1 cells after STEAP2 silencing. All data are presented as the mean ± standard deviation of three independent experiments. *P < 0.05, **P < 0.01, and ***P < 0.001.

Supplementary Figure S6. For the *in vivo* tumor metastasis assay, shSTEAP2 or sh-NC transfected K1 cells were injected subcutaneously into the flanks of the mice. Afterwards, tumor weight (a, b) in mice and growth curve of xenografts (c) were determined. For the *in vivo* tumor metastasis assay, indicated transfected K1 cells were injected into the tail vein of the mice following tail vein injection. (d) Representative images of metastatic tumor nodules in the lung of nude mice. (e) Number of metastatic tumor nodules in the lung were compared between nude mice injected with STEAP2-saRNA and NC-saRNA and statistically analyzed. All data are presented as the mean ± standard deviation. *P < 0.05, **P < 0.01, and ***P < 0.001.

Supplementary Figure S7: STEAP2 block hedgehog signaling pathway activation. (a) Endogenous SMO was immunoprecipitated from BCPAP cells with or without STEAP2 overexpression. Cellular extracts were immunoprecipitated with anti-SMO antibody, then immunoblotting analyses tested the serine-phosphorylation expression level. P-Ser, phosphorylated serine. (b) GLI1 nuclear translocation were evaluated by immunofluorescence staining in BCPAP cells. Nuclei were counterstained by DAPI. Bar, 20 μm

Supplementary Figure S8. The expression levels of STEAP2 in METTL3 knockdown PTC cells were detected by and western blot.

Supplementary Figure S9. STEAP2 mRNA level in METTL3-knockdown PTC cells treated with actinomycin D at the indicated time points were detected by

qRT-PCR. All data are presented as the mean ± standard deviation of three independent experiments. *P < 0.05, **P < 0.01, and ***P < 0.001.

Supplementary Figure S10. (a) Schematic presentation of proposed mutation site for disruption of catalytic site of METTL3 protein. (b) The expression of STEAP2 in BCPAP and TPC-1 cells transfected with empty vector (FLAG), wide type-METTL3 (METTL3-FLAG) or catalytic mutant METTL3 (METTL3-FLAG-Mutant) was analyzed by western blotting;

Supplementary Figure S11. The expression of STEAP2 in PTC cells transfected with si-YTHDF2 or si-NC was analyzed by western blotting;

Supplementary Fig. S12. The expression of STEAP2 in BCPAP and TPC-1 cells transfected with negative control (NC), METTL3 plasmid (METTL3) with si-NC or si-YTHDF1, was analyzed by western blotting;

Supplementary Fig. S13. BCPAP and TPC-1 cell lines were transfected with METTL3-overexpression (METTL3) plasmids and siRNA targeting STEAP2 (si-STEAP2) as indicated. (a) EdU assay were used to determine the viability of TPC-1 cells. (b) TUNEL assay were used to determine the apoptosis rate of TPC-1 cells. All data are presented as the mean ± standard deviation of three independent experiments. Scale bar: 50 μm. *P < 0.05, **P < 0.01, and ***P < 0.001.
