# Supplementary material for: METTL3-mediated m6A modification of STEAP2 mRNA inhibits papillary thyroid cancer progress by blocking the Hedgehog signaling pathway and epithelial-to-mesenchymal transition
Source: Cell Death Dis. 2022 Apr 18;13(4):358. doi: 10.1038/s41419-022-04817-6 (PMC9016063; doi:10.1038/s41419-022-04817-6)
Supplement: Supplementary file 2 — Supplementary table S1 [file 41419_2022_4817_MOESM2_ESM.docx]

**Table S1:The saRNA sequences used in the present study**

| sa-NC | 5’-ACUUACGUGACAGUAGA[dT][dT]-3’ |
| --- | --- |
| sa-STEAP2#1 | 5’-CTTAAGGGCCCACATGTGTT[dT][dT]-3’ |
| sa-STEAP2#2 | 5’-GGCGGATACTCAGCCGCTCG[dT][dT]-3’ |
